# Evaluating the anti-*Candida* effects of selenium nanoparticles impregnated in acrylic resins: An in vitro study

**DOI:** 10.34172/joddd.41113

**Published:** 2024-12-14

**Authors:** Masomeh Rostamzadeh, Seyyed Amin Sadeghi Sangdehi, Himen Salimizand, Bijan Nouri, Farshad Rahimi

**Affiliations:** ^1^Department of Prosthodontics, Faculty of Dentistry, Kurdistan University of Medical Sciences, Sanandaj, Iran; ^2^Department of Vaccinology and Immunotherapeutics, School of Public Health, University of Saskatchewan, Saskatoon, SK, Canada; ^3^Departement of Epidemiology & Biostatistics, Faculty of Medicine, Kurdistan University of Medical Sciences, Sanandaj, Iran

**Keywords:** Acrylic resin, Candida, Denture, Selenium nanoparticles

## Abstract

**Background.:**

*Candida albicans* in the oral cavity causes denture-induced stomatitis, but current treatments have side effects and contribute to drug resistance. Selenium nanoparticles (SeNPs) show promise as an antimicrobial agent, but their effectiveness against *C. albicans* is unknown and warrants further research.

**Methods.:**

Acrylic resins containing different concentrations of SeNPs (0.2, 2, and 10 g/mL) were formulated and evaluated against *C. albicans* isolates. The minimum inhibitory concentration (MIC) of SeNPs was determined, and a fungal biofilm was developed on acrylic samples. The quantity of biofilm was assessed using scanning electron microscopy (SEM) and optical density (OD) at 570 nm after staining with crystal violet. Statistical analysis was performed using STATA software, with Kruskal-Wallis and Mann-Whitney tests to establish significance (*P*<0.05).

**Results.:**

The MIC of SeNPs was 25%. The OD in the group with 10% SeNPs was 0.477 and 0.547 in the group with 0.2%. Kruskal-Wallis test results showed that at least two groups among those studied had significant differences (*P*=0.0273). In pairwise comparisons, the differences between all the groups were statistically significant (*P*=0.049). SEM analysis confirmed the destruction of *C. albicans* cell walls, leading to reduced colonization, with the 10% group showing the highest efficacy.

**Conclusion.:**

The study demonstrated that SeNPs are effective against *C. albicans* colonization when combined with acrylic resin. Specifically, SeNPs exhibited enhanced antifungal properties at a concentration of 10%. These findings confirm that SeNPs are a promising alternative to traditional antifungal agents for treating oral candidiasis and denture-induced stomatitis.

## Introduction

*Candida albicans* is an opportunistic polymorphic fungus that can cause reversible morphological changes between yeast and mycelium pathogens.^1^ This fungus is a part of the natural flora of the mouth. In 85% of cases, the cause of oral infection is *C. albicans*. Artificial materials such as acrylic prostheses, the conditions of the person’s immune system, dry mouth, the use of antibiotics and systemic corticosteroids, and poor hygiene are among the predisposing factors for candidal infections in the oral cavity.^2^ Acrylic resin is the primary material for replacing lost tissue in edentulous patients. However, microbial growth is observed under acrylic resin bases in patients over time.^3^ The use of removable prostheses increases the colonization of *Candida* in the oral cavity and causes complications such as stomatitis and angular cheilitis. Decreased oxygen in the vacuum conditions under dentures turns the harmless form of *C. albicans* into a pathogenic form, which can cause many complications and turn the oral cavity into a reservoir for systemic infections in these people.^4^ Dentures, as a foreign object, provide a favorable environment for the growth of microorganisms and can disturb the balance of the normal flora of the oral cavity.

 The adhesion and colonization of *Candida* on the physical surfaces of the denture lead to the production of microbial plaques on denture surfaces. Also, the interaction of *C. albicans* with acrylic resin increases the oral pH and creates favorable conditions for the growth of *C. albicans *and other microorganisms. The dissemination of denture-induced stomatitis through the mucosa can lead to *Candida*-disseminated infection in people with immune system deficiency.^5^ Since patients with complete dentures are mostly older adults, they may not have the physical ability to fully observe hygiene due to other diseases such as Parkinson’s or the ability to remember health points advised by the physicians due to Alzheimer’s disease.^4^ Although a dental implant is increasingly used in treating partial and completely edentulous patients, conventional partial and complete dentures made from polymethyl methacrylate (PMMA) are still the treatment of choice in many cases due to financial and medical issues.^6^ In this regard, 60% of people wearing removable prostheses have dental stomatitis, which has a multifactorial etiology. *C. albicans* is one of the most significant factors in this regard.^4^

 The primary attachment and adhesion of *C. albicans* to the tissue surface of the denture base and its colonization cause denture stomatitis. Mechanical cleaning methods alone are insufficient to reduce the level of microorganisms in dentures.^7^ Denture stomatitis treatment is difficult due to the incomplete disinfection of the acrylic surface and the rapid recolonization of the microbial agent.^8^ Despite using antifungal drugs for dental stomatitis treatment, the infection often continues, creating resistance to the *Candida* biofilm.^9^ Applying an antifungal agent to acrylic resin can play a vital role in preventing disease. The use of nanoparticles can be effective for the treatment of fungal infections. Owing to their unique properties, these particles can have more inhibitory power at a lower concentration than antibiotics.^10^

 Thanks to their unique physical, chemical, and electrical properties, selenium nanoparticles (SeNPs) have attracted the attention of many researchers in recent years. This quasi-metallic nanoparticle has also attracted the attention of researchers in this area as one of the significant nanoparticles in medical sciences due to its various biological properties, such as antioxidant, antimicrobial, anti-biofilm, and strengthening of the immune system. Studies conducted in recent years have shown the higher effectiveness and lower toxicity of SeNPs compared to other forms of selenium, such as selenite, selenomethionine, and selenocysteine.^11^ Selenium is a trace metal element that is vital as a nutrient and has crucial human health benefits due to its dynamic role in inhibiting the formation of free radicals. Therefore, selenium prevents oxidative stress, the primary source of age-related diseases. There are approximately 25 known selenoproteins in the human genome. Selenium enters yeast by chemisorption, with the formation of ionic bonds by cell wall polymers. The fungicidal effect may be due to the placement of selenium instead of sulfur (S) (due to the chemical analogy of both elements) in cellular proteins. Sulfur-containing amino acids such as cysteine (Cys) and methionine (Met) are changed. Using transporters such as Sul1 and Sul2 sulfate selenoproteins, yeasts transport selenium into the cytosol and generate reactive oxygen species that cause DNA strand breaks. This process can cause changes in protein misfolding, stability, structural changes, and enzyme dysfunction. The toxic activity of inorganic selenium compounds in yeasts includes the reaction of selenites with thiol-containing compounds. Thus, SeNPs have shown an anti-*Candida* effect.^11,12^ Hence, we investigated the inhibitory effect of acrylic resins containing SeNPs on *C. albicans* in Sanandaj, Iran. The null hypothesis for this study was that acrylic resins containing SeNPs would not have an inhibitory effect on *C. albicans*.

## Methods

 Since using various antimicrobial nanoparticles to prevent *C. albicans* from adhering to acrylic dentures is recommended,^11^ the present cross-sectional study was conducted at Kurdistan University of Medical Sciences in 2021. Twenty-five sterilized acrylic resin discs of the same size were examined, considering a 95% confidence level. Any pieces that were contaminated before the start of the experiment were excluded. SeNPs with a concentration of 1000 ppm were purchased as a solution and homogenized using an ultrasonic device before application. The American Type Culture Collection (ATCC) *C. albicans *isolates were obtained as a stock sample from the reference culture collection of the Tehran Medical Mycology Laboratory (TMML) and stored at -70 °C. It was then cultured on Sabouraud dextrose agar medium and maintained at 4‒6 °C throughout the experiment.

###  Acrylic resin nanoparticle disc preparation

 In this study, heat-cured acrylic resin dentures were used to create acrylic pieces containing SeNPs. Before commencing the work, all sterilizable devices were autoclaved for sterilization. The acrylic powder was also sterilized using gamma rays. Samples of control acrylic resin and those containing selenium with varying concentrations (n = 40) were randomly prepared using the standard press and pressure technique. Initially, a glass mold was used to create silicon patterns in the shape of discs measuring 4 × 6 mm. To ensure smooth and uniform samples, they were sandwiched between two glass surfaces before use. Four groups of acrylic resin pieces were prepared based on the concentration of SeNPs. To create the acrylic samples, 71 g of powder and 400 µL of monomer liquid were mixed according to the concentration of nanoparticles in each portion (0, 0.2, 2, and 10%). The selenium nanoparticle solution was added to the powder and monomer mixture. For example, a selenium nanoparticle solution with an 0.2% concentration required 13 µL of the 150 000 ppm nanoparticle solution. In comparison, a solution with a 2% concentration needed 133 µL of the 150 000 ppm nanoparticle solution, and a solution with a 10% concentration required 666 µL of the 150 000 ppm nanoparticle solution. The resulting compounds were mixed into silicone patterns and processed following the manufacturer’s instructions. The mixtures were then packed into molds. The acrylic resin-based denture was processed in the oven for 20 minutes at 20% power and then for 5 minutes at 90% power. The molds were air-dried, and the acrylic additions were trimmed using a sterile blade.

###  Determining the minimum inhibitory concentration (MIC)

 The antifungal effect of SeNPs was determined using the serial dilution method to establish the MIC in Mueller‒Hinton broth (Sigma Aldrich, Steinheim, Germany), following the protocol set by the Clinical and Laboratory Standards Institute. After creating serial dilutions in test tubes, they were incubated at 35 °C for 24 hours. The MIC was defined as the lowest concentration at which fungal growth was not observed.

###  Biofilm formation on resin discs

 Before the test, the acrylic pieces were cleaned ultrasonically in distilled water for 20 minutes. They were then exposed to ultraviolet light in a dry condition at room temperature for another 20 minutes to eliminate any microorganisms that could contaminate the samples. If contamination occurred during manufacturing or storage, the pieces were discarded.

**Figure 1:**
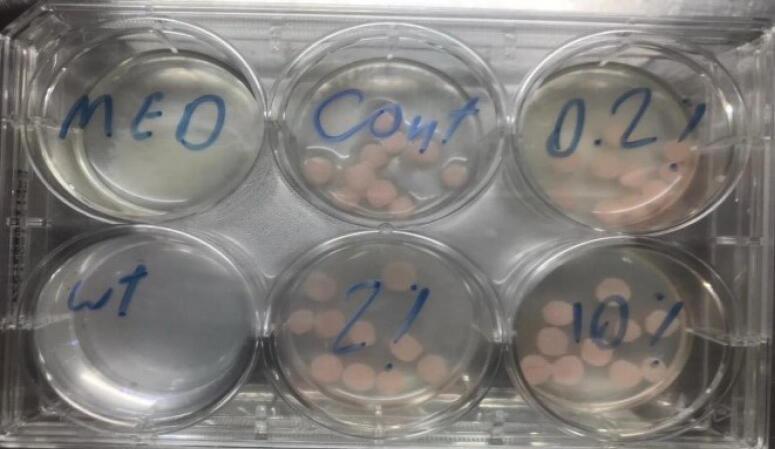
Formation of *Candida albicans* biofilms on acrylic resin pieces in a six-well microplate

 Subsequently, 3 mL of a standard suspension of *C. albicans* (ATCC10231) at a concentration of 1 × 10^3^ CFU/mL was added to 4 wells of 6-well microplates. Each well contained 10 acrylic pieces with varying concentrations (0.2%, 2%, and 10%) of SeNPs; 3 mL was also added to a well containing acrylic pieces without SeNPs. Additionally, one well contained only fungal suspension, and another contained distilled water (Figure 1).

 The plates were then placed in a shaker incubator for 24 hours at 37 ºC at 75 rpm. After incubation, the samples were gently washed twice with phosphate-buffered saline (PBS) to remove any cells not attached to the denture. They were then incubated for an additional 24 hours.

 Finally, the samples from each well were divided into two equal groups.

###  Biofilm assay by crystal violet staining

 The amount of *C. albicans* biofilm was measured in the first group of acrylic resin pieces using crystal violet staining. Each sample of prosthetic base acrylic resin containing nano-selenium was placed on the flat bottom of a 96-well microtiter plate. The acrylic samples were washed three times with 300 µL of PBS and then air-dried. Subsequently, 150 µL of methanol was added to the samples, which were placed in an incubator for one hour to fix (foil paper was wrapped around the plate to prevent light exposure). Following this, 150 µL of crystal violet was added to the samples. The samples were then washed with PBS to remove the unbound dye. Next, 100 µL of 95% ethanol was added to the wells for 30 seconds to completely dissolve the crystal violet absorbed by *C. albicans*. The optical density (OD) of crystal violet dissolved in the microplates was measured at 570 nm using a microplate reader (BIOTEK Synergy HT, Winooski, United States).

###  Biofilm assay by SEM 

 After incubating the samples for 24 hours, the second group of samples after fixation was examined under a scanning electron microscope (SEM) with an acceleration voltage of 20 kV to measure the amount of *C. albicans* on the surface.

###  Statistical analysis

 The data were analyzed using STATA-12 software. The significance level was set at 0.05. The non-parametric Kruskal-Wallis test was used to compare the three groups. The results showed that at least two studied groups had significant differences. To determine the differences between the groups, pairwise comparisons were made using the non-parametric Mann-Whitney test. For all tests, a *P* value of < 0.05 was considered statistically significant.

## Results

###  MIC and crystal violet staining analysis

**Figure 2:**
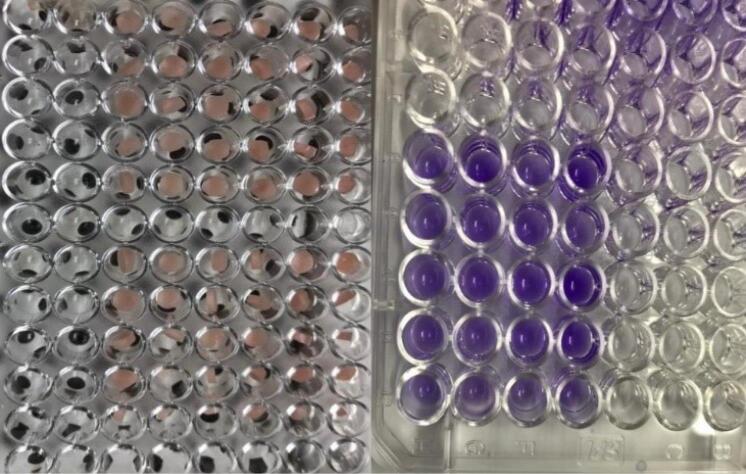
Crystal violet staining of acrylic pieces in a 96-well microplate

 The fungal population was reduced by 99.9% in 50% (500 ppm) and 25% (250 ppm) concentrations of SeNPs. The culture medium with a concentration of 12.5% of SeNPs was also not turbid, but its transparency was less than two concentrations above (Figure 2). Hence, the concentration of 25% was reported as the MIC of SeNPs against *C. albicans*.

**Figure 3:**
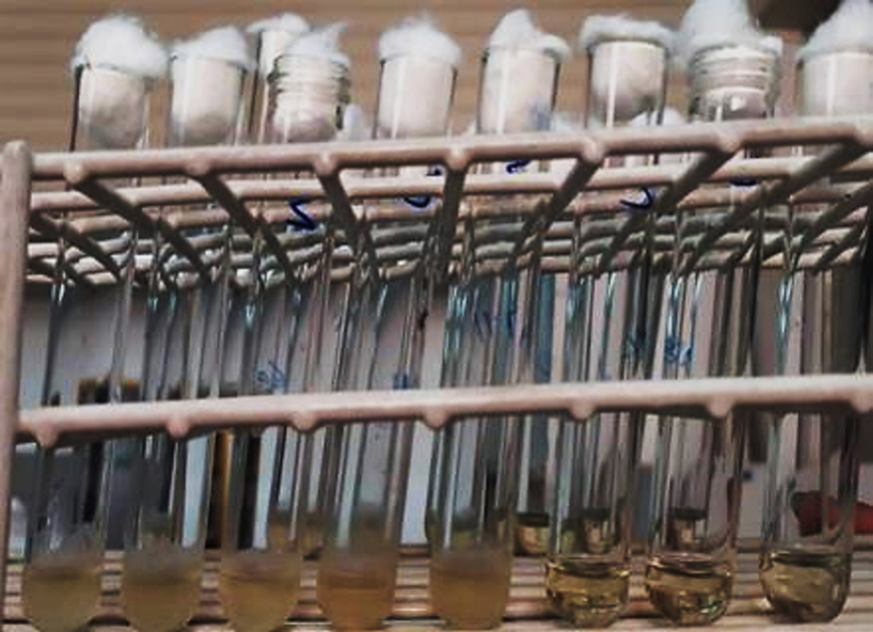
The minimum inhibitory concentration of selenium nanoparticles on *Candida albicans*. From left to right, there is a decrease in the turbidity of the environment

**Figure 4:**
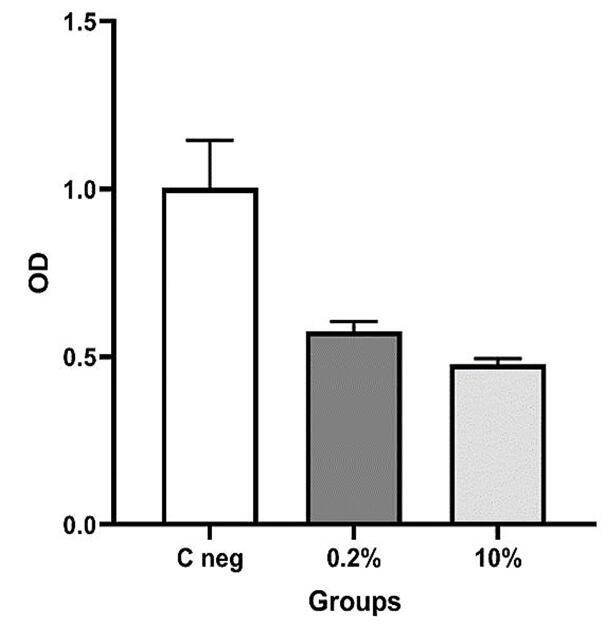
The amount of *C. albicans* in three groups of acrylic pieces with zero, 0.2, and 10% selenium nanoparticles based on OD

 The OD diagram of the samples by the microplate reader device showed that the lowest absorption rate is at a concentration of 10% of SeNPs. The OD of the samples increased with decreasing nanoparticle concentration (Figure 3). These numbers were statistically significant (Figure 4).

###  Data analysis

 According to the non-parametric Kruskal-Wallis test presented in Table 1, the results showed that at least two groups had significant differences (*P* = 0.0273).

**Table 1:** Mean, standard deviation, and number of fungal colonies

**Group**	**Mean**	**SD**	**n**
Control	1.003	0.141	3
0.2% concentration	0.547	0.029	3
10% concentration	0.477	0.018	3

Note: Kruskal-Wallis test; *P* value = 0.0273 significant.

 Using the non-parametric Mann-Whitney test, the groups were compared pairwise, and the results showed significant differences between all the studied groups (Table 2).

**Table 2:** Mann-Whitney test results

**Groups**	***P*** ** value**	
Control group and 0.2% concentration	0.049	Significant
Control group and 10% concentration	0.049	Significant
0.2% concentration and 10% concentration	0.049	Significant

###  SEM analysis

**Figure 5:**
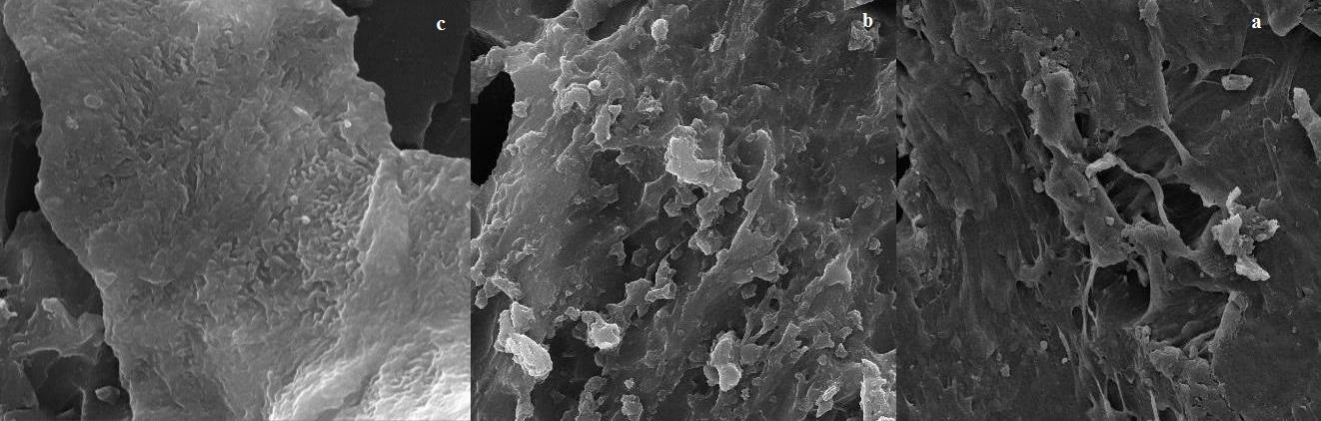
SEM analysis with × 100000 magnifications, (a) 10% and (b) 0.2% concentrations of selenium nanoparticles, and (c) control group

 Electron microscope images showing the effect of SeNPs in control groups and groups with 0.2% and 10% of SeNPs on biofilm formation showed that biofilm formation on acrylic resin pieces decreased with increasing concentrations of SeNPs (Figure 5).

## Discussion

 The most common treatment for oral reconstruction of edentulous patients is to fabricate a removable prosthesis from PMMA.^13^ In patients with complete dentures, oral candidiasis, and denture-induced stomatitis are the most frequent diseases caused by complete dentures (especially in the maxilla).^10^ Since the current local treatments for candidiasis are nystatin, fluconazole, and amphotericin B in more severe cases, issues such as the side effects of using these drugs and the development of drug resistance should also be considered for these types of local treatments.^14^

 Given what was stated, if an antimicrobial substance can be mixed with the acrylic resin during the preparation of the prosthesis, the incidence of the mentioned problems will decrease. Denture stomatitis treatment is difficult due to the incomplete disinfection of the acrylic surface and rapid recolonization of the microbial agent. Despite using antifungal drugs for denture stomatitis treatment, the infection often continues, and resistance against *Candida* biofilms develops.^7^ Thus, many efforts have been made to reduce *C. albicans* adhesion and colonization on the denture base using antifungal agents. One approach is to combine an antifungal agent with PMMA. Recently, nanoparticles have attracted considerable attention to control biofilm growth.^7^

 Selenium is one of the vital elements involved in many biological processes in different organisms through interaction with selenoproteins. It can also play an effective role in reproduction. It is possible to prevent many problems caused by the lack of this element, which can affect the health of a person, by providing adequate amounts of selenium in the diet. Nano-selenium has high bioavailability and low toxicity. Also, it can be a suitable supplement for different species if reasonably priced. Also, this nanoparticle is a better alternative than other types of salt. In the present study, conducted to investigate the inhibitory effect of SeNPs in acrylic resins on *C. albicans*, acrylic pieces with 10% SeNPs had the best antifungal effect. Based on the Kruskal–Wallis test, this effect was significant compared to the control group and 0.2% SeNP (*P* = 0.0273). The results of the Mann-Whitney test also showed a significant difference when the three studied groups were compared (*P* = 0.049). Electron microscope images also showed the highest antifungal effect of SeNPs at a concentration of 10%, and the antifungal effect was lower at 0.2% concentration.

 Abuhajar et al^7^ noted that nanoparticles such as silver, titanium dioxide, zinc oxide, and zirconium dioxide exhibit antimicrobial properties. Silver nanoparticles can damage cell membranes and can be incorporated into PMMA to decrease colonization by *C. albicans*. Similarly, the presence of TiO_2_ and halloysite clay in PMMA has also been shown to reduce C. albicans colonization. While coating dentures with antimicrobial substances can prevent *C. albicans* adhesion, it is important to note that this may impact denture retention and increase microbial colonization if the coating is damaged.

 Li et al^15^ investigated the effect of denture base acrylic resins containing silver nanoparticles on the attachment and formation of *C. albicans* biofilm. Resin bases containing silver nanoparticles were effective against *C. albicans* only in high concentrations (5%).

 Ghahramanloo et al^10^ investigated the antifungal effect of silver nanoparticles in acrylic resins. The antifungal effect increased with increasing contact time and concentration of silver nanoparticles in acrylic resin containing silver nanoparticles. An unfavorable result from the integration of silver nanoparticles with acrylic resin was the increase in the opacity of PMMA.

 Sharma et al^16^ investigated three metal nanoparticles of gold, silver, and platinum. The potential dangers of silver nanoparticles and their toxicity for body organs and cells were evaluated more than the other two nanoparticles. They reported that it is necessary to consider more precautions when using it. In many cases, gold nanoparticles were in the non-toxic range, and platinum nanoparticles were somewhere between these two poles in terms of toxicity.

 Based on the results of the present study and the properties of SeNPs,^17,18^ such as antioxidant, effective in improving learning, increasing hair growth, antibacterial properties, helping in food digestion, anti-cancer agent, moderator of the immune system, improving reproductive performance, and improving growth performance, this nanoparticle can be an appropriate option to combine with PMMA to prevent *C. albicans* biofilm formation.

 Shurygina et al^19^ showed that in the form of nano-sized particles, elemental Se is not only biocompatible but also has some biological activities (antitumor, antimicrobial, and protective). The biological properties of SeNPs, such as toxicity, selectivity for various types of cells, biocompatibility, and biodegradability, directly depend on their physical and chemical properties. Also, it has been demonstrated that Se in the nanoscale form has a dose-dependent effect. High concentrations of SeNPs ( > 2 mg Se/kg) can cause Se toxicity in mammals.

 Ryabova et al^20^ reported no specific toxic action attributable solely to selenium. The LOAEL and NOAEL values are contradictory. The NOAEL was 0.22 mg/kg body weight per day for males and 0.33 mg/kg body weight per day for females, while the LOAEL was assumed to be a dose of 0.05 mg/kg of SeNP.

 Lara et al reported the inhibitory effect of SeNPs in a dose-dependent manner on *C. albicans* biofilm with IC_50_ at 21.7 ppm. Chitosan alone had a 7% inhibitory effect on biofilm at 25 ppm. CS-SeNPs showed the strongest inhibitory effect against biofilms formed in a dose-response manner with IC_50_ at 3.5 ppm, indicating a strong synergistic effect compared to both compounds alone. They also compared the activities of CS-SeNPs on the biofilms of two drug-sensitive strains (SC5314 and TW1) and two drug-resistant *C. albicans* strains (TW17 and 6486). They reported no significant difference between the drug-sensitive and drug-resistant strains in the dose-response curve (*P* < 0.05).^21^

 In a study by Cheraghi Saray et al,^22^ *Lactobacillus* species were used to produce nano-selenium-enriched cell mass. Their results revealed the highest non-growth zone for the well and disc methods, with 29.41 and 27.64 mm, respectively, belonging to the nano-selenium treatment loaded in lactobacilli on the standard *C. albicans* species. The examination of MIC and MFC showed that the treatments of nanoselenium loaded in lactobacilli and nanoselenium + *Lactobacillus* had the lowest mean for both tests. They reported that the results of microbial tests confirm the high antifungal power of nano-selenium treatment loaded in lactobacilli.

 Given what was stated, it is necessary to find an approach that prevents the adhesion of the fungus to the acrylic resin. One of these approaches is combining an antifungal agent with PMMA. Thus, many efforts have been made to reduce *C. albicans* adhesion and colonization on the denture base using antifungal agents. Recently, biocidal nanoparticles have attracted considerable attention for controlling the growth of biofilm.^7^

## Conclusion

 Based on several studies, many nanomaterials have a positive impact on inhibiting and controlling the growth of oral candidiasis. However, by comparing the effects of these nanomaterials, given their benefits and harms for humans, and since nano-selenium has high bioavailability and low toxicity and has a reasonable price, as an option, it is considered appropriate for mixing with denture base material to prevent *C. albicans* biofilm formation. Considering the limitations, such as ethical responsibilities, time-consuming nature, and lack of access to clinical strains, the present study was conducted on the standard *C. albicans* strain and acrylic resin pieces in a laboratory environment. To achieve more objective and clinical results, further studies on clinical strains and dentures of patients (with their consent) are recommended.

## Acknowledgments

 The study was sponsored by the Department of Research and Technology of Kurdistan University of Medical Science, Sanandaj, Iran.

## Competing Interests

 The authors declare that no known competing financial interests or personal relationships that could have influenced the work reported in this paper.

## Ethical Approval

 The protocol of this study was approved by the Ethics Committee of the Kurdistan University of Medical Sciences (Ethical approval code: IR.MUK.REC.1400.193).
